# Transitional Infrastructures Extending Access to Safe Water in Informal Settlements: A Cross-Sectional Study in Nairobi, Kenya

**DOI:** 10.4269/ajtmh.24-0108

**Published:** 2024-10-22

**Authors:** Vitor Pessoa Colombo, Jürg Utzinger, Fred Orina, Michael Otieno, Hellen Meme, Jérôme Chenal

**Affiliations:** ^1^ École Polytechnique Fédérale de Lausanne, Lausanne, Switzerland;; ^2^ Swiss Tropical and Public Health Institute, Allschwil, Switzerland;; ^3^ University of Basel, Basel, Switzerland;; ^4^ Kenya Medical Research Institute, Nairobi, Kenya;; ^5^ Nairobi City County Government, Nairobi, Kenya;; ^6^ CEAT, École Polytechnique Fédérale de Lausanne, Lausanne, Switzerland;; ^7^ Mohammed VI Polytechnic University, Ben Guerir, Morocco

## Abstract

There is a pressing need for transitional water infrastructures in rapidly growing cities where conventional infrastructures lag behind human settlement expansion. In Kenya, sectoral innovations have addressed local socioeconomic challenges, but empirical evidence on their efficacy (combining quantitative indicators of safety and continuity) is lacking. We addressed this gap by comparing different water infrastructures in their ability to provide constant access to safe water in informal settlements in Nairobi, Kenya. We conducted a cross-sectional survey including 1,147 households in two informal settlements. Water infrastructures were categorized based on their distribution system: 1) piped to premises; 2) piped to a neighboring compound; 3) public tap/dispenser; and 4) street vendor. We tested associations between these systems with two outcomes: constant water availability and diarrhea (stratified by age group). We used adjusted odds ratios (AORs) to test associations between distribution systems and the selected outcomes, while accounting for confounders. Obtaining water from public taps/dispensers or street vendors was associated with service continuity (AOR = 1.45, 95% confidence interval [CI]: 1.06–1.99; AOR = 11.16, 95% CI: 2.45–50.82). Piped sources were associated with service disruption, especially when obtained from a neighboring compound (AOR = 0.45, 95% CI: 0.28–0.70). Public taps/dispensers were the only system consistently associated with lower odds of diarrhea, notably in children under the age of 5 years (AOR = 0.47, 95% CI: 0.29–0.79). Hence, in cities with a high prevalence of informal settlements and limited financial resources, public taps and dispensers hold promise as transitional water infrastructures.

## INTRODUCTION

Universal access to safe drinking water is anchored in two of the United Nations’ Sustainable Development Goals (SDGs); namely, in SDG 6 (water and sanitation) and in SDG 11 (sustainable cities and communities). However, achieving this fundamental right by 2030, as aspired to by the SDGs, remains a formidable challenge, especially in low- and middle-income countries (LMICs). According to the World Health Organization (WHO) and United Nations Children’s Fund (UNICEF) Joint Monitoring Program for Water Supply, Sanitation, and Hygiene (JMP), in 2020, an estimated 2 billion people lacked access to “safely managed” water services, including about 800 million people that did not have access even to a “basic” drinking water source.^1^ The JMP defines “safely managed” water services as facilities providing “drinking water from an improved source that is accessible on premises, available when needed, and free from fecal and priority chemical contamination.”^1^ As for “basic” drinking water services, the JMP defines them as facilities providing “drinking water from an improved source, provided collection time is not more than 30 minutes for a round trip, including queuing.”^1^

The JMP alerts that global progress rates, as observed between 2015 and 2020, need to increase by a factor of 4 to achieve SDG target 6.1 (universal access to “safely managed” water services). This slow pace might be explained by the lack of financial resources. By the time the SDGs were launched, a World Bank study^2^ pointed out that if global investments on water, sanitation, and hygiene (WASH) remained the same as in the period between 2000 and 2015, target 6.1 would not be achieved by 2030. In fact, the authors emphasized that this target required tripling the global annual investments of the previous period and, in the case of developing regions, it was even a challenge to afford universal coverage of lower service levels, i.e., “basic” water services.

Globally, emphasis has been placed on capital-intensive systems, such as city-wide piped water networks, considered a “technological paradigm” directly associated with the idea of development.^3^^,^^4^ However, given the relatively high construction and maintenance costs of piped water infrastructures,^5^ their implementation in LMICs constitutes a challenge and has led to fragmented cities. For instance, in many parts of Africa, cities are historically marked by a spatial concentration of urban infrastructures, including adequate water services and facilities, failing to reach the poorest populations that are pushed to “informal” settlements.^6^^,^^7^ The latter are defined by the United Nations Human Settlements Program (UN-Habitat) as “residential areas where inhabitants are deemed by the authorities to have no legal claim to the land they occupy and the system of occupation ranges from squatting to informal rental housing.”^8^ Such disparities in access to adequate housing and infrastructures has affected cities across the world, fostering significant urban health inequities,^9^^–^^11^ which became even more evident during the COVID-19 pandemic.^12^

**Figure 1.**
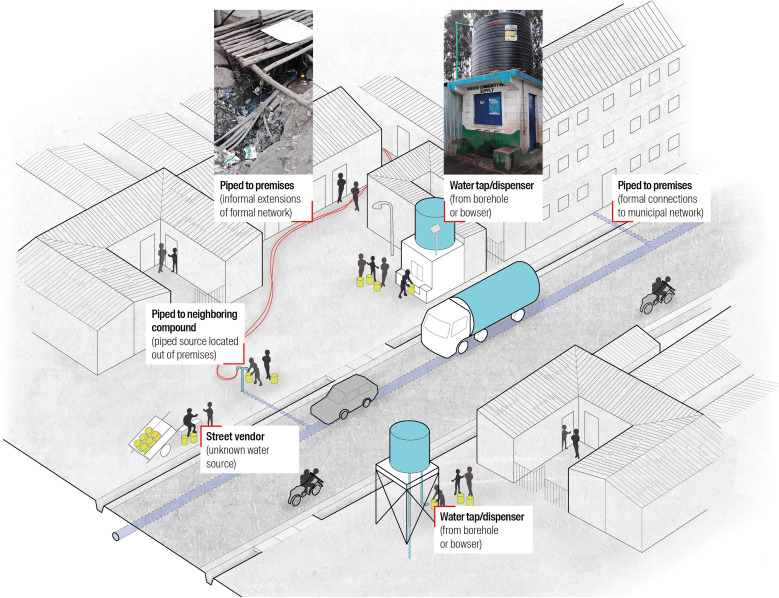
Diverse designs and distribution systems of water services in informal settlements. Drawing modified by the authors; pictures by Geoffrey Mboya and Vitor Pessoa Colombo.

Given the current challenges to provide, in the short-term, universal access to “safely managed” and even “basic” water services, governments in LMICs should consider transitional solutions that are both affordable and safe. Sectoral innovations in African cities have fostered promising, alternative water services through new infrastructure designs, but the latter still lack empirical assessments combining safety and continuity indicators. In this regard, assessing the ability of decentralized (nonpiped) water distribution systems that emerged in the last decade is particularly relevant. For instance, public water dispensers, also referred to as water “ATMs,” with water sourced from boreholes or bowsers and stored in large containers, have been implemented in large cities like Nairobi^13^ and Freetown.^14^ The adaptation of these facilities to the socioeconomic contexts of informal settlements has been discussed and even contested by several qualitative studies.^15^^–^^17^ However, except for a few studies in other countries,^14^^,^^18^^,^^19^ there is a paucity of empirical, quantitative evidence on these recent water services. More specifically, there is a lack of studies comparing the abilities of different water services to provide a continuous service and prevent diarrheal diseases based on their design and distribution system (Figure 1), instead of relying solely on the conventional “improved”/“unimproved” categories.

We addressed this gap by assessing how different water distribution systems performed in two informal settlements in Nairobi based on their associations with two self-reported outcomes: 1) water availability as an indicator of service continuity and accessibility, and 2) diarrhea, as a proxy indicator of safety. On the one hand, diarrheal diseases remain among the main causes of death worldwide, with an estimated total of more than 1.6 million deaths in 2016, of which approximately half were directly attributed to the use of unsafe WASH services.^20^ Notably, this burden disproportionally affects sub-Saharan Africa, where the mortality rate attributed to diarrhea was 61.8 per 10,000 in 2016, the highest among all regions.^21^ On the other hand, sufficient access to water impacts key hygiene habits such as handwashing^22^ and is crucial to reduce the risk of diarrheal diseases.^23^

## MATERIALS AND METHODS

### Geographical scope.

**Figure 2.**
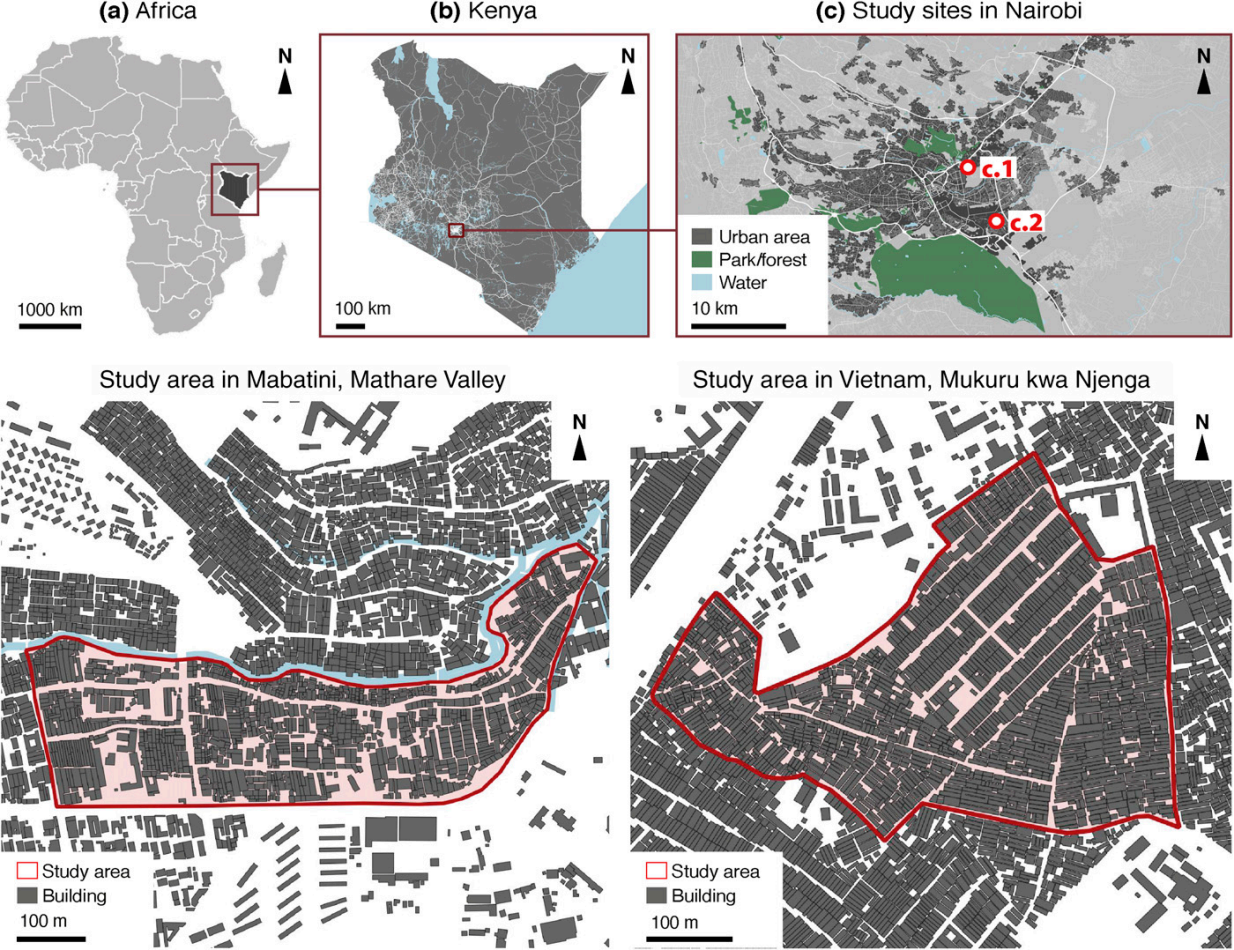
Location of selected study sites in Nairobi, Kenya. Modified from GADM, OpenStreetMap, Ecopia Building Footprints © 2021 Ecopia Tech Corporation, Imagery © 2021 DigitalGlobe, Inc.

Our study focused on two informal settlements in Nairobi, Kenya (Figure 2). Nairobi is a relevant study site given the diversity of technologies used to deliver water to low-income areas, ranging from piped networks to independent water vendors and shared standpipes. Since the creation of the Informal Settlements Department in 2009, the Nairobi City Water and Sewerage Company (NCWSC) has elaborated innovative strategies to expand “formal” services to low-income areas through a varied “menu” of systems.^24^ In this context, decentralized systems have emerged, from independent water vendors to the water “ATMs” provided since 2015 by the NCWSC. Moreover, the coverage of at least “basic” water sources in Kenyan cities has stagnated at 87%, while access to “safely managed” water services slightly decreased from 61% in 2000 to 58% in 2020.^25^ Understanding the potential health benefits of alternative, low-cost water distribution systems might be a critical contribution to address the urgent need to expand these services in Nairobi and in other settings facing similar challenges.

In 2019, 70% of the population in Nairobi was estimated to live in informal settlements.^26^ This study focused on such settlements because they illustrate the contemporary challenges in extending access to adequate WASH services to low-income, urban populations in LMICs.^27^ Hence, we selected the following study sites: 1) Mabatini, located in the Mathare Valley, and 2) Vietnam, located in Mukuru kwa Njenga. The former is situated in the northeastern part of the city, along the Mathare River, whereas the latter is in the southern part of the city, near a large industrial area. The selection was governed by the presence of a variety of water distribution systems, from private, piped connections to public taps, dispensers, and street vendors.

### Study design and definitions.

The study design was elaborated through a collaboration between researchers in urban planning and public health and the Nairobi City County Government. We designed a cross-sectional survey and collected primary household data in two informal settlements in Nairobi through a structured questionnaire. Water distribution systems (Figure 1) were categorized into four types: 1) water piped to the household’s premises, including informal extensions from the municipal network; 2) water piped to a neighboring compound, also including informal extensions from the municipal network; 3) public water taps or dispensers, i.e., a nonpiped system with a large storage capacity, including water “ATMs”; and 4) street vendors.

We considered the household’s primary drinking water source as the reference to determine which system would be attributed to each household. We emphasized the distinction between water piped to the household’s premises (the first type) from water piped to a neighboring compound (the second type), because the latter is often part of informal water reselling schemes,^16^^,^^17^ being more similar to street vendors than to water piped to premises. The key difference between “water piped to a household’s premises” and “water piped to a neighboring compound” is that in the latter, people need to fetch water from a third party outside their compound and, hence, have less control of and access to the source.

The four categories above fall into the JMP’s definition of “improved” water sources. Of relevance, this definition focuses on the means used to deliver water to the consumer, without addressing its chemical properties and quality (e.g., presence of bacteria or other pathogens), which was not assessed in this study. Moreover, our categorization deliberately avoids the “formal”/“informal” dichotomy, as the distinction between these two sectors is often inaccurate, given their intertwined economical and spatial transactions.^28^ Hence, our categorization focused on the design and distribution system of water services, regardless of their status.

### Outcomes of interest.

The primary outcome was self-reported diarrhea with a recall period of 2 weeks. Diarrhea is defined as the passage of three or more loose or liquid stools within 24 hours^29^ and has been used by multiple studies as a reference outcome to estimate the quality of WASH services.^30^^,^^31^ The second outcome of interest was the availability of drinking water over a 30-day period, self-reported by the participants through a questionnaire. This was based on the JMP’s 2018 update on core questions on WASH for household surveys, which recommends addressing the continuity of access to water by inquiring whether there had been “any time when the household did not have sufficient quantities of drinking water when needed in the month preceding the survey.”^32^

### Data collection and inclusion criteria.

We collected primary data between July and August 2021 through structured household questionnaires conducted by a team of 18 enumerators. We chose this period to avoid the rainy season, which could impose logistic issues to enter the selected sites. Enumerators followed a 4-day training prior to the survey to become familiarized with the questionnaire and with the mobile data collection tools used, that is, the application KoboCollect 1.30.1 (Kobo, Cambridge, MA) operated in tablets (Galaxy Tab A 8.0 2019 [Samsung, Suwon-si, South Korea]).

We calculated the minimum sample size based on the expected prevalence of diarrhea among children under the age of 5 years. The calculation details are given in the Supplemental Material. The minimum sample size for both sites was 1,138 households; hence, we aimed for a minimum of 569 surveys in each site. The two study sites were subdivided into survey areas with an equivalent count of buildings detected from aerial images. The same number of household surveys was conducted in each survey area to ensure a homogenous spatial distribution of data collection. The enumerators were instructed to apply the “random walk” method^33^ to select households in their respective survey area. Individuals aged 18 years or older were targeted by the questionnaire, and preference was given to heads of households.

## STATISTICAL ANALYSES

To test associations between diarrhea and the four water distribution systems, we used adjusted odds ratios (AORs) stratified by age (general population and children under the age of 5 years). The four types of water distribution systems were the exposures of interest. Several control variables (Table 1) were selected from a preliminary list of candidate features (see Supplemental Material), all of which had been associated with diarrhea in other studies.^34^^,^^35^ To account for external contamination sources, we also included the frequency of street food consumption in the list of candidate control variables. The six control variables that were included in the multiple logistic regressions (MLRs) were selected based on bivariate analyses (Supplemental Table 1). Only variables having a *P*-value <0.1 were included in the MLRs. This analysis used individual-level data, as diarrhea cases were reported individually for each household member.

**Table 1:** Variables included in the MLRs to test associations between self-reported diarrhea and water distribution systems in two informal settlements in Nairobi, Kenya, in July and August 2021

Dependent Variable	Stratification	Selected Covariates
Diarrhea: whether the individual had diarrhea (self-reported) in the 2 weeks preceding the survey (0 = no; 1 = yes)	General population: *N*_gen pop_ = 3,174 individuals* in the general population (two sites combined) Under-five stratum: *N*_under 5_ = 410 individuals* in the population under 5 years of age (two sites combined)	Exposure: type of distribution system delivering water to the individual’s household (main drinking water source), with 1 MLR run separately for each of the four types, as they are mutually exclusive (0 = given type is not used; 1 = given type is used) Control 1: individual lives in a household that regularly treats drinking water before drinking it, with chlorine tablets or bleach (0 = no; 1 = yes) Control 2: the individual’s household had access to sufficient water in the month preceding the survey, whenever needed (0 = no; 1 = yes) Control 3: individual lives in a household considered overcrowded^†^ (0 = no; 1 = yes) Control 4: individual lives in a relatively wealthy household (0 = no; 1 = yes) Control 5: the head of the household where the individual lives accomplished secondary education (0 = no; 1 = yes) Control 6: individual lives in a household where consumption of street food is frequent (0 = no; 1 = yes)

MLRs = multiple logistic regressions.

* *N_x_* is the number of individuals living in a household with valid answers for all eight variables used in the MLRs.

^†^ More than three inhabitants per room, as defined by Sustainable Development Goal 11.^36^

To assess the ability of each system to ensure constant access to water, we used unadjusted odds ratios (ORs) as well as AORs. We used the latter to account for socioeconomic variables (Table 2) that might affect the household’s ability to purchase water in sufficient quantities, which could lead to water scarcity independently of the chosen water system. In contrast to the previous analysis, the MLRs were based on household-level data, as water availability was reported for the entire household.

**Table 2:** Variables included in the MLRs to test associations between self-reported water availability and distribution systems in two informal settlements of Nairobi, Kenya, in July and August 2021

Dependent Variable	Selected Covariates
Water availability: whether the household had sufficient quantities of drinking water when needed, throughout the month preceding the survey (self-reported) (0 = no; 1 = yes)	Exposure: type of distribution system delivering water to the individual’s household (main drinking water source), with 1 MLR run separately for each of the 4 types, as they are mutually exclusive (0 = given type is not used; 1 = given type is used) Control 1: individual lives in a household considered overcrowded* (0 = no; 1 = yes) Control 2: the head of the household where the individual lives accomplished secondary education (0 = no; 1 = yes) Control 3: individual lives in a relatively wealthy household (0 = no; 1 = yes)

MLRs = multiple logistic regressions.

* More than three inhabitants per room, as defined by Sustainable Development Goal 11.^36^

For both outcomes, an association was considered statistically significant if it met the following conditions: 1) the AOR’s 95% confidence interval (CI) excluded 1, and 2) the logistic model’s overall fit was acceptable, i.e., a likelihood ratio test’s (LLR) *P*-value was lower than 0.05. We checked for multicollinearity between the variables included in the MLRs by calculating their variance inflation factors (VIFs). To be included in the model, a variable needed to have a VIF lower than or equal to 5. Further details are given in the Supplemental Material. Because we had four mutually exclusive exposure variables, for each outcome we ran four separate MLRs, i.e., one for each water distribution system.

## RESULTS

### Participants and types of water services identified.

**Figure 3.**
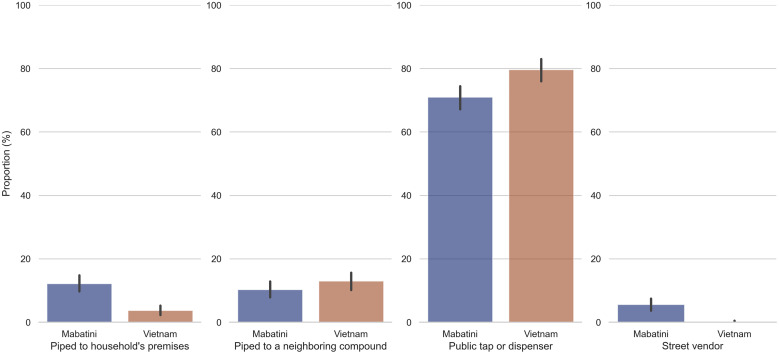
Water distribution systems used in two informal settlements of Nairobi, Kenya, according to household data collected between July and August 2021 (black lines indicate 95% CIs of each observed proportion).

Overall, 1,147 households were surveyed, 576 in Mabatini and 571 in Vietnam, thus meeting the minimum sample size requirements. This corresponded to 3,786 individuals, 1,935 in Mabatini and 1,851 in Vietnam. Among these, 2,011 participants were female (53%), and 491 (13%) were children under the age of 5 years. Nearly all households (99%) had access to what the JMP defines as “at least basic” water services. Among these, 75% obtained drinking water mainly from a public tap/dispenser, 8% had water piped to their household’s premises, 12% obtained water from a piped source at a neighboring compound, and 3% obtained water mainly from street vendors. Figure 3 summarizes the use of water distribution systems in each site.

### Relations between water distribution systems, diarrhea, and service continuity.

The prevalence of diarrhea in the general population was 12.12% (95% CI: 10.99–13.26%), with 13.71% (95% CI: 12.05–15.37%) in Mabatini and 10.41% (95% CI: 8.88–11.94%) in Vietnam. For children younger than 5 years, the observed prevalence was 26% (95% CI: 22.31–30.86%), with 27.46% (95% CI: 21.16–33.76%) in Mabatini and 25.81% (95% CI: 19.98–31.63%) in Vietnam. Obtaining drinking water mainly from a public tap or dispenser was significantly associated with lower odds of self-reported diarrhea in children below the age of 5 years than those obtaining water from other sources (Table 3). Conversely, obtaining water mainly from a piped source located at a neighboring compound was significantly associated with higher odds of self-reported diarrhea in this age group. The other distribution systems did not show any significant association or trend for diarrhea.

**Table 3:** AORs for self-reported diarrhea by type of water distribution system in two informal settlements of Nairobi, Kenya, in July and August 2021

Distribution System	General Population (*N* = 3,174*)				Children under the Age of 5 Years (*N* = 410^†^)			
	AOR	Lower 95% CI	Upper 95% CI	Significance	AOR	Lower 95% CI	Upper 95% CI	Significance
Water piped to premises	1.17	0.80	1.71	Not significant	1.64	0.72	3.75	Not significant
Water piped to a neighboring compound	0.90	0.64	1.27	Not significant	**1.99**	**1.06**	**3.74**	** ^†^
Public tap or dispenser	0.90	0.70	1.16	Not significant	**0.47**	**0.29**	**0.79**	*** ^†^
Street vendor	1.82	0.73	4.51	Not significant	0.84	0.08	8.60	Not significant

AORs = adjusted odds ratios; MLRs = multiple logistic regressions. Bold indicates statistically significant results.

* Corresponds to the number of individuals living in a household with valid answers for all eight variables (dependent, exposure, and control variables).

^†^ The number of asterisks indicates the significance of each beta coefficient resulting from the MLR, which corresponds to the probability of the AOR being equal to 1: ** *P*-value <0.05; *** *P*-value <0.01.

When water availability was assessed (Table 4), overall, only 36.68% of households (95% CI: 33.89–39.47%) reported that they never lacked water during the month preceding the survey. Water availability was higher in Vietnam (40.70%, 95% CI: 36.67–44.73%) than in Mabatini (32.70%, 95% CI: 28.86–36.53%). Street vendors and public taps/dispensers showed a consistent, positive association with service continuity, while piped water sources were consistently associated with service disruption, i.e., one or more episodes of lack of water in the month preceding the survey. Obtaining water from street vendors was the mode of distribution most strongly associated with self-reported water availability. Both the unadjusted and adjusted ORs showed a significant, positive association. Regarding public taps/dispensers, the unadjusted OR was not statistically significant, but the AOR showed a significant association with water availability. As for water obtained mainly from a piped source in a neighboring compound, it was consistently and significantly associated with service disruption, in contrast to street vendors and public taps/dispensers.

**Table 4:** Unadjusted ORs and AORs for the self-reported availability of water by distribution system in two informal settlements of Nairobi, Kenya, in July and August 2021

Distribution System	Unadjusted ORs (*N* = 1,143*)				Adjusted ORs (*N* = 1,027^†^)			
	OR	Lower 95% CI	Upper 95% CI	Significance	AOR	Lower 95% CI	Upper 95% CI	Significance
Water piped to premises	0.94	0.60	1.46	Not significant (LLR *P*-value ≥0.05)	0.96	0.60	1.55	Not significant (LLR *P*-value ≥0.05)
Water piped to a neighboring compound	**0.42**	**0.27**	**0.66**	**** ^‡^	**0.45**	**0.28**	**0.70**	**** ^‡^
Public tap or dispenser	1.22	0.92	1.63	Not significant (LLR *P*-value ≥0.05)	**1.45**	**1.06**	**1.99**	** ^‡^
Street vendor	**12.94**	**4.51**	**37.16**	**** ^‡^	**11.16**	**2.45**	**50.82**	*** ^‡^

AORs = adjusted odds ratios; LLR = likelihood ratio; MLR = multiple logistic regression; ORs = odds ratios. Bold indicates statistically significant results.

* Corresponds to the number of households with valid answers for the two variables (dependent and exposure variables).

^†^ Corresponds to the number of households with valid answers for all five variables (dependent, exposure, and control variables).

^‡^ The number of asterisks indicates the significance of each beta coefficient resulting from the MLR, which corresponds to the probability of the AOR being equal to 1: ** *P*-value <0.05; *** *P*-value <0.01; **** *P*-value <0.001.

Of relevance, the two outcomes of interest were significantly correlated, which is why water availability was included in the list of control variables in Table 1. Individuals living in a household with constant water availability throughout the month preceding the survey had lower odds of self-reported diarrhea, both in the general population and in children younger than 5 years (respectively, OR: 0.53, 95% CI: 0.42–0.67, and OR: 0.55, 95% CI: 0.35–0.85). When adjustments were by the other control variables correlated with diarrhea listed in Table 1, the AORs were 0.53 (95% CI: 0.41–0.68) for the general population and 0.66 (95% CI: 0.40–1.08) for children under the age of 5 years.

## DISCUSSION

### Assessing distribution systems based on water availability and risk of diarrhea.

In terms of water availability, households obtaining water mainly from street vendors or public taps/dispensers were at higher odds of having constant access to water than households with piped connections. This finding was confirmed by the descriptive statistics aggregated by site. More households reported benefiting from a continuous water service in Vietnam (40.70%) than in Mabatini (32.70%). In the former, a higher proportion of households relied on water taps/dispensers than in the latter (Figure 3). The better service continuity of street vendors and water taps/dispensers might be explained by the flexibility and capillarity of such sources, which can be mobile (in the case of vendors) and provide water on demand in customized quantities. Of note, although the AOR for water availability from street vendors was statistically significant, it featured a wide 95% CI. This might be explained by the low number of households (*N* = 33) obtaining water from street vendors as a primary drinking water source.

Nairobi is chronically affected by water shortages that result in disruptions in water delivery through piped networks and disproportionally affect the poorest areas.^37^ In this context, the ability to store water is crucial to ensure the continuity of water services.^38^ It is precisely this resilience against water shortages that may explain why we observed higher odds of water availability in water taps/dispensers and independent water vendors than in piped networks. Indeed, piped systems rely on constant water delivery and are, hence, more prone to service disruption in situations of water stress. In contrast, water taps/dispensers and street vendors rely on storage systems and do not depend on a centralized network.

In terms of water safety, estimated by the associated risk of self-reported diarrhea, for children below the age of 5 years, consuming water mainly from a public tap or dispenser was significantly safer than obtaining water from other sources. These results are in line with a recent study in urban slums in Malawi, which found that water from public water taps had a higher probability of having a “safe” concentration of *Escherichia coli*, i.e., near zero per 100 mL, than other sources.^19^ However, we note that the safety of drinking water obtained from public taps also depends on the use of safe containers, i.e., containers that prevent the introduction of external objects (and, hence, contamination) when water is carried or stored.^31^^,^^39^ Previous studies in Nairobi have reported the use of unsafe water containers by informal water vendors.^17^ Moreover, purchasing water from street vendors instead of more conventional sources has been reported to increase water costs in Nairobi’s informal settlement.^17^^,^^40^ In this sense, albeit more reliable in terms of service continuity, obtaining water mainly from street vendors might actually result in water rationing within the household for economic reasons, with negative impacts on hygiene habits.

Our findings suggest an interrelation between the continuity of water services and the risk of diarrhea. The higher odds of diarrhea observed when water is obtained from a piped source at a neighboring compound might be explained by the chronic lack of water associated with this distribution mode. Conversely, the lower odds of diarrhea observed in water obtained from public taps/dispensers might be related to their ability to ensure the continuity of water services. In fact, water shortages may impact key hygiene habits (e.g., handwashing), which has been reported elsewhere.^22^

We did not observe any statistically significant association between self-reported diarrhea and access to “improved” sanitation either in the general population (OR: 0.95, 95% CI: 0.78–1.18) or in children under the age of 5 years (OR: 1.10, 95% CI: 0.72–1.68). The same holds for self-reported diarrhea and the presence of basic hygiene facilities in the household, both for the general population (OR: 0.91, 95% CI: 0.58–1.41) and for children under the age of 5 years (OR: 1.92, 95% CI: 0.69–5.38). This is why these variables were not included in the MLRs as control variables, although they can be determinants for the risk of diarrhea.^31^ In the case of improved sanitation, the wide 95% CI might be explained by the lack of information on the quality and maintenance of facilities; for instance, whether and how latrines are regularly emptied. As for basic hygiene, a large proportion of households (538 out of 1,147) did not allow the surveyors to observe the presence of handwashing amenities, namely, the availability of soap and water in their dwellings. This might have led to a selection bias affecting the resulting ORs between basic hygiene services and self-reported diarrhea.

### Most common water distribution system.

Public water taps/dispensers were the most frequently used systems in the two study sites in Nairobi (Figure 3). Indeed, approximately three-quarters of the participating households reported using them as the main source of drinking water. This is in line with the 2019 Kenya Population and Housing Census,^41^ which presented public water taps and standpipes as the main drinking water sources in urban informal settlements.

The implementation of public water taps/dispensers aimed at regulating water prices in informal settlements renders them more affordable through direct provision of water by the NCWSC. This is not the case, for instance, for independent reselling schemes (e.g., street vendors or domestic water reselling). However, as Sarkar showed in recent studies,^13^^,^^16^ this economic advantage depends on the management of water points. The author explains that although distributers are usually accredited by the public utility, in some cases water from public dispensers and taps may be resold extralegally by independent vendors who charge higher prices than those practiced by the public utility.

### Policy recommendations and research needs.

Our study resonates with previous observations on the practical limitations to provide, in the short and medium term, drinking water through centralized piped systems in LMICs.^15^^,^^42^^,^^43^ When transitional solutions to expand water services through low-cost interventions are assessed, there is a balance to be found between safety and availability of water. Our findings suggest that in the context of limited resources and chronic water scarcity, the continuity of water services was more likely to be guaranteed through decentralized distribution systems, such as water dispensers or street vendors. The latter, however, tended to be associated with higher odds of diarrhea in the general population, albeit not significantly. Public water taps and dispensers, on the other hand, performed significantly well both in terms of service continuity and water quality.

From a design perspective, systems offering water storage capacity seem more resilient, particularly in low-income areas where water shortages are common. Moreover, with an adequate governance framework to regulate tariffs, infrastructures like public water taps/dispensers could increase the affordability of this essential service. Indeed, they have the potential to ensure the safety and continuity of water services where piped networks are not available or affordable in the short term.

From a monitoring perspective, we found that some types of “basic” water sources as defined by the JMP are not necessarily reliable in terms of service continuity. In fact, households using water from public taps, dispensers, or street vendors were more likely to have constant access to water than those obtaining water from a piped source. This confirms the necessity of including indicators of both safety and availability of water in the JMP water service ladder, as is the case in the “safely managed” category and as recommended by the JMP in their 2018 update on core questions on WASH for household surveys.^32^

From a governance perspective, the lack of a framework adapted to the decentralized water provision currently in place in Nairobi might negatively impact the quality of water obtained from independent vendors, including public taps/dispensers and water reselling from piped connections. Although sectoral reforms brought by the 2002 Water Act expanded access to water by including independent vendors as service providers, these reforms have failed to provide performance indicators of “pro-poor” interventions and, particularly, to enforce quality standards.^16^^,^^17^

More research is needed to confirm our findings, notably, through cluster randomized trials focusing on interventions on water services and employing microbiological assessments. Still, in view of the empirical evidence generated, we argue that effective provision of water services in cities with stark differences in socioeconomic and spatial configurations such as Nairobi requires a variety of infrastructures that match these intraurban differences. This includes transitional solutions that are implementable in the short term, until “safely managed” services can be universally introduced. Most importantly, there is no one-size-fits-all solution.

### Study limitations.

The cross-sectional nature of this study is a limitation. Although this design was useful to explore associations between different water distribution systems and a selection of outcomes, no causal association could be established. In the case of diarrhea, no etiologic inference could be made, as we did not collect any biologic sample and did not perform any microbiological water quality assessment, as this was beyond the scope of this exploratory study. Notably, the lack of water quality assessment prevented us from establishing certain associations between each water source and the risk of diarrhea, as contamination may happen between the source and the point of use.

Besides the water quality at the source, there are relevant environmental exposures that could explain differences in the risk of diarrhea, but these were not addressed in this study. Notably, these include cohabitation with animals^44^ and, for children under the age of 5 years, the quality of outdoor playing areas, which might increase exposure to pathogens.^45^ In fact, the risk of diarrhea varies spatially,^11^ and, in some contexts, it might be more significantly associated with features of the urban landscape than with WASH services.^10^

Moreover, self-reporting of diarrhea and water availability makes our study susceptible to reporting biases, as participants may not always remember the occurrence of these outcomes with the same accuracy. To mitigate these limitations, we included control variables related to socioeconomic status, which have been related to recall biases regarding diarrheal cases.^34^ Another factor that might have biased our data is the data collection period, for two reasons. First, data collection took place while the Kenyan government implemented preventive measures against COVID-19, generally increasing access to water services. Second, data collection during the dry season might have reduced the overall probability of diarrheal cases, as the incidence of diarrhea is associated with heavy rainfall.^46^

## Supplemental Materials

10.4269/ajtmh.24-0108 Supplemental Materials
