# Supplementary material for: Transitional Infrastructures Extending Access to Safe Water in Informal Settlements: A Cross-Sectional Study in Nairobi, Kenya
Source: Am J Trop Med Hyg. 2024 Oct 22;112(1):208–16. doi: 10.4269/ajtmh.24-0108 (PMC11720795; doi:10.4269/ajtmh.24-0108)
Supplement: Supplemental Materials [file tpmd240108.SD1.pdf]

Supplementary materials - Transitional infrastructures extending access to safe water in informal settlements: a cross-sectional study in Nairobi, Kenya

SAMPLE SIZE

The sample size was determined by the expected prevalence of the primary outcome[47]; in our case, diarrhea. We used the following formula, applicable to prevalence studies:

$$n_0 = \frac{(Z_{1-\propto/2}) \times P \times (1-P) \times D_{eff}}{e^2}$$

where $\boldsymbol{n_0}$ is the number of people required for the survey; $\boldsymbol{Z}_{\boldsymbol{1-\propto/2}}$ is the critical value for the standard normal distribution corresponding to a type I error rate of **α** (here, **α** = 0.05, hence, $\boldsymbol{Z}_{\boldsymbol{1-\propto/2}}$ = 1.96); $\boldsymbol{P}$ is the expected prevalence rate of the outcome of interest in the population of interest; $\boldsymbol{D_{eff}}$ is the "design effect" of cluster sampling; and $\boldsymbol{e}$ is the margin of error to be tolerated at 95% level of confidence (here, $\boldsymbol{e}$ = 0.05). The prevalence of diarrhea is usually higher among children under the age of 5 years than among older individuals. Hence, we used the expected prevalence amongst children in this age group as the parameter $\boldsymbol{P}$ to ensure that the sample size was sufficiently large. We set the expected diarrheal prevalence at 20%, based on a previous study covering different informal settlements in Nairobi[48]. The same study was used as a reference to establish the design effect parameter ($\boldsymbol{D_{eff}}$ = 1.50).

The sample size ($\boldsymbol{n_0}$) corresponds to an estimated number of people needed for the survey. Given that the unit of analysis in our study was the household, sample size calculation needed to be adjusted and counted in terms of households. On this basis, the initial sample ($\boldsymbol{n_0}$) was adjusted by: (i) the proportion of the targeted population (% of children younger than 5 years in the general population, estimated at 12%); (ii) the average household size (number of people, estimated at 3 individuals); and (iii) the expected valid response rate (here, 90%). Demographic data were obtained from the 2019 Kenya Population and Housing Census[49]. Following these parameters, the minimum sample size was 1,138 households.

SELECTION OF CONTROL VARIABLES (*AOR*s FOR DIARRHEA)

To calculate the AORs for self-reported diarrhea, we started by establishing a list of potentially significant variables that, based on the existing literature, could be relevant. The list included:

- **"Water available":** the individual lives in a household where water was available throughout the month preceding the survey (self-reported)
- **"Improved sanitation":** the individual has access to improved sanitation (observed)

- **"Basic hygiene":** the individual lives in a household equipped with a hand-washing facility, with soap and water available at the moment of the survey (observed)
- **"Use of water recipient (*any*)":** the individual lives in a household where drinking water is obtained from a source out of premises and stored in a recipient of *any* type (self-reported)
- **"Use of water recipient (*closed*)":** the individual lives in a household where drinking water is obtained from a source out of premises and stored in a *closed* recipient (self-reported)
- **"Water treatment":** the individual lives in a household where drinking water is treated with bleach, chlorine, or boiled (self-reported)
- **"Overcrowding":** the individual lives in a household with more than 3 inhabitants per room (self-reported)
- **"Frequent street food consumption":** the individual eats street food twice or more per week, on average (self-reported)
- **"Secondary education":** the individual lives in a household where the person in charge (head of household) completed secondary education (self-reported)
- **"Relatively wealthy household":** the individual lives in a household with more goods than the average (based on a pre-established list of goods, self-reported)

The six control variables listed in Table 1 were selected based on preliminary, bivariate analyses between each candidate feature above and the outcome of interest (see Table 5 below). Only variables having a *P*-value <0.1 (purposively "loose" at this preliminary stage) were included as control variables in the MLRs. In addition, we only considered ORs obtained from logistic models with an overall fit that was acceptable, i.e., a LLR *P*-value smaller than 0.05. We recurred to this pre-selection of control variables to increase the power of our models by avoiding saturating the MLRs with too many variables.

Table 5. Unadjusted odds ratios (ORs) for self-reported diarrhea, by candidate control variable in two informal settlements of Nairobi, Kenya, in July and August 2021.

Candidate covariate	Subset	Unadjusted OR	Lower 95% CI	Upper 95% CI	Significance (*P*-value)[a]
Water available	children under five	0,55	0,35	0,85	***
	general population	0,53	0,42	0,67	****
Improved sanitation	children under five	1,10	0,72	1,68	Not significant (LLR *P*-value ≥ 5%)
	general population	0,96	0,78	1,18	Not significant (LLR *P*-value ≥ 5%)

Basic hygiene	children under five	1,92	0,69	5,38	Not significant (LLR *P*-value ≥ 5%)
	general population	0,91	0,58	1,41	Not significant (LLR *P*-value ≥ 5%)
Use of water recipient (*any*)	children under five	0,94	0,44	2,00	Not significant (LLR *P*-value ≥ 5%)
	general population	1,02	0,71	1,47	Not significant (LLR *P*-value ≥ 5%)
Use of water recipient (*closed*)	children under five	1,42	0,87	2,30	Not significant LLR *P*-value ≥ 5%)
	general population	0,90	0,72	1,12	Not significant (LLR *P*-value ≥ 5%)
Water treatment	children under five	1,11	0,74	1,66	Not significant (LLR *P*-value ≥ 5%)
	general population	0,81	0,66	0,99	**
Overcrowding	children under five	1,59	1,03	2,46	**
	general population	1,14	0,93	1,39	Not significant (LLR *P*-value ≥ 5%)
Frequent street food consumption	children under five	1,68	1,05	2,70	**
	general population	1,62	1,28	2,05	****
Secondary education	children under five	0,57	0,37	0,88	**
	general population	0,84	0,68	1,03	Not significant (LLR *P*-value ≥ 5%)
Relatively wealthy household	children under five	0,58	0,38	0,87	***
	general population	0,83	0,68	1,01	Not significant (LLR *P*-value ≥ 5%)

[a] The number of * indicates the significance of each *beta* coefficient resulting from the MLR, which corresponds to the probability of the AOR being equal to 1: **P*-value <0.1, ***P*-value <0.05, ****P*-value <0.01, *****P*-value <0.001.

DATA AND CODE

The coding materials (in Python) and data processing steps used in the statistical analyses can be found here: https://github.com/ceat-epfl/water-informal-settlements

REFERENCES

47. Lwanga SK, Lemeshow S., 1991. Sample size determination in health studies: a

practical manual. Geneva: World Health Organization

48. African Population and Health Research Center., 2014. Population and health dynamics in Nairobi's informal settlements: Report of the Nairobi Cross-sectional Slums Survey (NCSS) 2012. Nairobi: African Population and Health Research Center

49. Government of Kenya., 2019. 2019 Kenya Population and Housing Census Volume III - distribution of population by age, sex and administrative units. Nairobi: Kenya National Bureau of Statistics
